# The energy‐based scaling of a thin current sheet: Case study

**DOI:** 10.1002/2015GL066189

**Published:** 2015-11-19

**Authors:** Yu. L. Sasunov, M. L. Khodachenko, I. I. Alexeev, E. S. Belenkaya, E. I. Gordeev, I. V. Kubyshkin

**Affiliations:** ^1^ Space Research InstituteAustrian Academy of SciencesGrazAustria; ^2^ Skobeltsyn Institute of Nuclear PhysicsFederal State Budget Educational Institution of Higher Education M.V.Lomonosov Moscow State UniversityMoscowRussia; ^3^ Earth Physics DepartmentSaint Petersburg State UniversitySaint PetersburgRussia

**Keywords:** Cluster observation, adiabatic invariant, current sheet

## Abstract

The influence of average plasma energy $\tilde{E}$ on the half thickness *ℓ* of a thin current sheet (TCS) is investigated for three cases of TCSs crossings. The value of *ℓ* was estimated from the magnetic field data by means of Cluster observations. The obtained scaling values for TCSs, $\tilde{Z}=\ell /\rho_{T}$, where *ρ*_*T*_ is the thermal Larmor radius, were compared with the scaling $Z_{\mu }=2\sqrt{2\tilde{E}/T}$, where $\tilde{E}$ and *T* are the average plasma energy and the temperature of plasma, which assumes a specific dynamics (conservation of magnetic flux through the trajectory segment) of the current carriers. The comparison of $\tilde{Z}$ and *Z*_*μ*_ shows a good agreement.

## Key Points

- The new scaling is presented
- The energy‐based scaling compared with observable thickness of TCS
- The population of the TCS‐forming protons results in different scaling

## 1 Introduction

The TCSs play an important role in space plasma physics. In particular, they are crucial building blocks of various astrophysical phenomena, such as magnetospheres of planets, current systems of solar and stellar flares, and heliospheric and astrospheric large‐scale current layers. TCSs are closely related with processes of energy exchange between magnetic field and surrounding plasma. These processes usually occur inside the TCS characterized by thickness *λ* = 2*ℓ* which could be considered as self‐thickness of a TCS. The information about *ℓ* (or *λ*) could be used for the estimation of accumulated (or released) energy in a TCS [*Parker*, 1963; *Furth et al.*, 1963].

The TCSs in the Earth's magnetosphere have been extensively investigated in the last decades by means of space missions (e.g., Time History of Events and Macroscale Interactions during Substorms, Cluster, etc.). For instance, the spacecraft measurements in the Earth's magnetotail show that the thickness of the embedded TCSs can be a few hundred kilometers before substorm onset [*Mitchell et al.*, 1990; *Sergeev et al.*, 1993], which is comparable with the thermal Larmor radius *ρ*_*T*_=*V*_*T*_/*ω*_*L*_ of protons, where *ω*_*L*_ is the Larmor frequency of proton gyration and $V_{T}=\sqrt{\mathrm{kT}/m_{i}}$ is proton's thermal speed. The statistical studies of magnetotail TCS crossings showed that *ℓ* can have a rather wide range: *ρ*_*T*_<*ℓ* < 20*ρ*_*T*_ [*Runov et al.*, 2005]. Moreover, *ℓ* also depends on the topology of TCS, such as bifurcation or asymmetry [*Runov et al.*, 2006]. For further study, we introduce a so‐called magnetic scaling $\tilde{Z}=\ell /\rho_{T}$, where the term “magnetic” is used to indicate that the parameter *ℓ* is calculated from the magnetic field data measured by spacecraft.

One of the first attempts to understand the kinetic nature of TCSs showed that formation of a TCS could be related with motion of transiting protons on Speiser orbits [*Eastwood*, 1972]. Further theoretical development showed that in vicinity of a TCS, different kinds of proton trajectories could be distinguished: transient, cucumber, and ring types [*Büchner and Zelenyi*, 1989]. Later, a few models of TCSs were proposed where the electric current is mainly formed by special population of transient protons, whereas particles with cucumber and ring orbits are less efficient [*Kropotkin and Domrin*, 1996; *Zelenyi et al.*, 2000]. Within the frames of the so far proposed models, a strong condition on the proton's motion was imposed, namely the approximate conservation of adiabatic invariant $I_{z}=\oint V_{z}dz\approx$ const, where *z* axis is codirected with the normal to the TCS. This condition leads to the fact that the TCS theoretical thickness *ℓ* ^∗^ has a range of 0 < *ℓ* ^∗^≤*ρ*_*T*_ [*Sitnov et al.*, 2000], and the corresponding scaling of the TCS is a function of the ratio of particle thermal *V*_*T*_ to the incoming flow *V*_*D*_ velocities: *Z*_*I*_=*ℓ* ^∗^/*ρ*_*T*_=*F*(*V*_*T*_/*V*_*D*_)*Zelenyi et al.* [2000].

Quite often, there is a disagreement between the theoretical estimation of the TCS thickness obtained with *I*_*z*_= const assumption (i.e., *ℓ* ^∗^), and that given by spacecraft measurements: *ρ*_*T*_<*ℓ* < 20*ρ*_*T*_ [*Runov et al.*, 2005, 2006]. This disagreement is resolved by renormalizing of scaling, i.e., by considering the geometrical constraints imposed on the magnetic field around the TCS [*Artemyev et al.*, 2010]. In particular, introducing the magnetic field *B*_ext_ in the far region of the TCS which is higher than the magnetic field *B*_0_ in close vicinity (characterized by 2*ℓ* ^∗^) enables to correct the observable scaling *ℓ*/*ρ*_*T*_ by multiplying it by a small factor *k* ^2^=(*B*_0_/*B*_ext_)^2^, which could be considered as a renormalization coefficient. Based on statistical observations, it has been shown that the reasonable value of *k* is ∼0.4 [*Artemyev et al.*, 2010].

On the other hand, if one impose another condition on the dynamics of protons, namely the conservation of magnetic flux through a segment of trajectory (*μ* = const), then the scaling of a TCS becomes dependent on the ratio of the particle incoming flow *V*_*D*_ to thermal *V*_*T*_ velocities, i.e., $Z_{\mu }=F^{\prime }(V_{D}/V_{T})$, which could be expressed as follows [*Sasunov et al.*, 2015]:

(1) $$Z_{\mu }=2\sqrt{2\tilde{E}/T}.$$

Here *T* and $\tilde{E}$ are the temperature (expressed in energy units) and average energy of plasma, respectively. The last is expressed via 1‐D energy distribution function *f*(*E*):

(2) $$\tilde{E}=\overset{E_{\text{end}}}{\underset{0}{\int }}f(E)EdE/\overset{E_{\text{end}}}{\underset{0}{\int }}f(E)dE.$$

As *f*(*E*) we use here 1D particle energy flux obtained from the Cluster data. Therefore, the two TCS scalings, *Z*_*I*_ and *Z*_*μ*_, could be distinguished for different dynamic regimes of the TCS‐forming protons, which correspond to *I*_*z*_= const and *μ*= const conditions, respectively. For instance, if the observed TCS is mostly created by protons population with *I*_*z*_= const, the scaling has to be *ℓ*/*ρ*_*T*_≤1 (without renormalization), whereas for the *μ*= const condition, the measured scaling *ℓ*/*ρ*_*T*_, according to equation (1), becomes ≥1 (because $\tilde{E}\geq T$). Therefore, there is a possibility to resolve the above mentioned contradiction without renormalization of scaling just by assuming different TCS‐forming populations in different cases.

The goal of this work is to compare the TCS magnetic $\tilde{Z}=\ell /\rho_{T}$ and *Z*_*μ*_ (equation (1)) scalings using Cluster observations.

## 2 Cases Study

In our study, we use proton temperature *T* and distribution functions *f*(*E*) from the Cluster Ion Spectrometry (CIS) instrument [*Reme et al.*, 2001], as well as magnetic field measurements from the FluxGate Magnetometer (FGM) instrument [*Balogh et al.*, 2001]. The measured physical vectors and coordinates are in the GSE system, at the same time the TCS crossing process is considered in local system of coordinate (LMN). To transform the GSE data to a LMN system, we use the Minimum Variance Analysis method [*Sonnerup and Cahill*, 1967] and determine the **L,M,N** vectors of the LMN system as follows: **N** is normal direction to the TCS, *L* is the direction of maximally varying magnetic field, and **M** = [**N** × **L**].

The information about *ℓ* could be obtained from the consideration of the local electric current *j*_*M*_ as a function of the running coordinate *z* (along **N**), where the local electric current is given as *j*_*M*_(*z*) = *d* *B*_*L*_/d*z*. In this case, the half thickness *ℓ* is usually defined as a distance between two points at which *j*_*M*_ reaches the value of 0.5max(*j*_*M*_(*z*)). The calculation of *z* is based on a simple equation $z=\overset{t_{1}}{\underset{t_{0}}{\int }}V_{N}(\tau )\text{d}\tau$, where *V*_*N*_ is the normal plasma velocity whereas *t*_0_ and *t*_1_ are the start time and end time of integration, respectively. Obviously, the assumptions regarding the local normal velocity *V*_*N*_ have strong influence on the calculation of *z*, and nowadays there exist several experimental methods to define *V*_*N*_. Unfortunately, due to different restricting factors none of these methods can give precise estimates for *ℓ*. That is the reason why we use in our study two methods of determining *V*_*N*_ simultaneously.

The first method is the so‐called “timing” method, in which the local relative velocity *V*_*N*_ is defined as a ratio of the distance between two satellites to the delay of the TCS crossing time. This technique is widely used to determine the geometry of plasma processes [see, for example, *Sergeev et al.*, 2004]. Hereafter, we denote a TCS thickness calculated by this method as *ℓ*_tim_. The second method, called as the “reconstruction” method, is based on the reconstruction of the gradient ∇_*N*_ *B*_*L*_ along the normal vector direction. In this case the normal component of velocity is defined as *V*_*N*_=(d*B*_*L*_/d*t*)/(∇_*N*_ *B*_*L*_), where ∇_*N*_ *B*_*L*_ is restored from the satellite measurements [*Runov et al.*, 2005]. We denote the thickness of a TCS calculated with the reconstruction method as *ℓ*_rec_.

In the following subsections we present three examples of TCS crossings observed by Cluster, which were selected from the list in *Runov et al.* [2005] using the following criteria.

1. The magnetic field *x* component (in GSE) changes the sign during the crossing, and the magnetic field magnitude before and after the TCS is of the same order. This condition means that the satellites do not remain inside the TCS.
2. The plasma bulk velocity is less than 200 km/s, in order to avoid active periods of magnetotail dynamics.
3. The whole energy distribution function (1‐D energy spectrum) remains in the measurement range of CIS instrument. That is important for the correct calculation of $\tilde{E}$.
4. Two methods (“timing” and “reconstruction”) give comparable values of TCS half thickness with a maximum discrepancy <2*ρ*_*T*_.

### 2.1 Case 1

**Figure 1:**
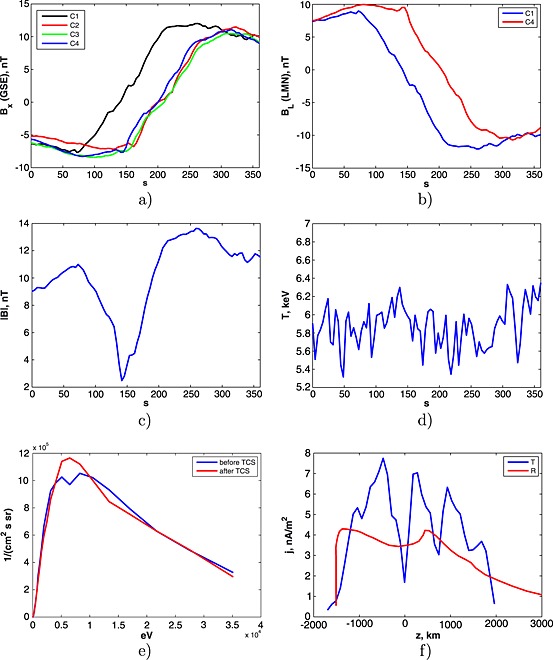
Cluster observations at 01:22:00/01:28:00 UT on 30 October 2001: (a) magnetic field (*x* component) measured by C1, C2, C3, and C4 satellites; (b) *B*_*L*_ components calculated for C1 and C4; (c) the total magnitude of magnetic field seen by C1; (d) plasma temperature according to C1 measurements; (e) 1‐D energy spectrogram measured by C1 before and after TCS; (f) the recalculated electric current profiles calculated by timing (blue line) and reconstruction (red line) methods.

Case 1 has start time/end time at 01:22:00/01:28:00 UT on 30 October 2001. All satellites, C1, C2, C3, and C4, observed a typical for the TCS crossing behavior of *B*_*x*_ (in GSE), i.e., the change of sign (see Figure 1a). For definiteness, we use the data provided by the satellites C1 and C4. Note that the recalculation for other satellites gives a similar result. The calculated **LMN** vectors are **L** = [−0.9345,0.3543,0.0343], **M** = [0.1370,0.2693,0.9533], **N** = [0.3285,0.8955,−0.3002] for the C1 and **L** = [−0.9176,0.3884,0.0843], **M** = [0.1874,0.2360,0.9535], **N** = [0.3505,0.8908,0.2894] for the C4. The behavior of the *B*_*L*_ components (for both satellites) is presented in Figure 1b.

The distribution of electric currents, calculated by two methods, are presented in Figure 1f. As expected, two different methods give different distributions of electric current along the running coordinate. The estimation of *ℓ* at the level of 0.5max(*j*_*M*_(*z*)) gives *ℓ*_tim_=2700 km and *ℓ*_*rec*_=3500 km. For the calculation of the magnetic scaling $\tilde{Z}=\ell /\rho_{T}$, it is necessary to compute the thermal Larmor radius, which is defined as $\rho_{T}[1000\;\text{km}]=3.25\sqrt{T[\text{keV}]}/|B|[\text{nT}]$. In fact, the measured values |*B*| and *T* could be different before and after the TCS crossing. In the considered case |*B*|=[|*B*|^before^,|*B*|^after^] = [10.9,12.9] nT, and *T* = [*T* ^before^,*T* ^after^] = [5.8,5.8] keV (see Figures 1c and 1d). Therefore, the Larmor radius has also different values (before/after): $\rho_{T}=[\rho_{T}^{\text{before}},\rho_{T}^{\text{after}}]=[720,600]$ km.

Since we do not know which of two methods for the estimation of *ℓ* is more precise in each particular case, we use in further calculation an average value for the TCS half thickness <*ℓ* >= (*ℓ*_tim_+*ℓ*_rec_)/2. Similarly, to simplify the treatment, also an average value for the Larmor radius $\rho =(\rho_{T}^{\text{before}}+\rho_{T}^{\text{after}})/2$ is considered; and finally, the average magnetic scaling may be defined as $<\tilde{Z}>=<\ell >/\rho$. In the considered case we obtain $<\tilde{Z}>=4.65$.

Based on the observed 1‐D energy distribution functions *f*(*E*), the average energy $\tilde{E}$ (equation (2)) was calculated numerically using a trapezoidal method. Note that $\tilde{E}$ could be also different before and after TCS because of some difference between the measured distribution functions on both sides of the TCS (see, for example, Figure 1e). In the considered case we have $\tilde{E}=[{\tilde{E}}^{\text{before}},{\tilde{E}}^{\text{after}}]=[15.1,\;14.9]$ keV.

The reason why we distinguish between $\tilde{E}$ values on both sides of the TCS is that corresponding populations of protons (before and after the TCS) equally contribute the creation process of the main electric current in the TCS. In view of that, the total *μ* scaling of the TCS could be expressed by simple averaging $<Z_{\mu }>=(Z_{\mu }^{\text{before}}+Z_{\mu }^{\text{after}})/2$.

In the considered case the scaling is <*Z*_*μ*_>=4.6. That is rather close to the corresponding $<\tilde{Z}>$ obtained above.

### 2.2 Case 2

**Figure 2:**
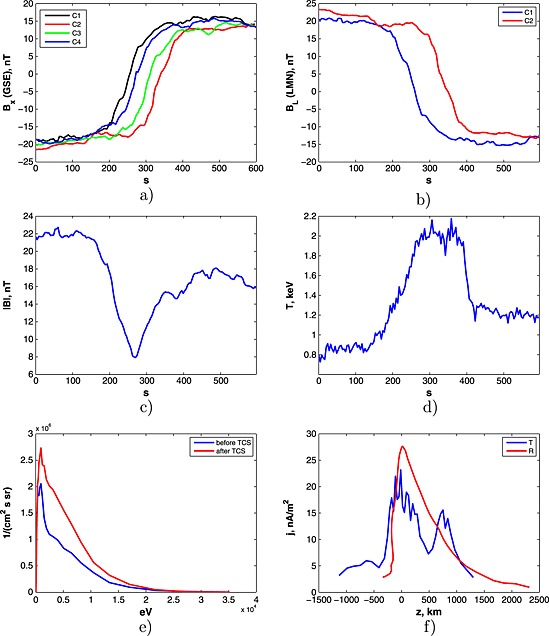
Cluster observations at 22:50:00/23:00:00 UT on 14 September 2001: (a) magnetic field (*x* component) measured by C1, C2, C3, and C4 satellites; (b) *B*_*L*_ components calculated for C1 and C2; (c) the total magnitude of magnetic field seen by C1; (d) plasma temperature according to C1 measurements; (e) 1‐D energy spectrogram measured by C1 before and after TCS; and (f) the recalculated electric current profiles calculated by timing (blue line) and reconstruction (red line) methods.

The case 2 has start time/end time at 22:50:00/23:00:00 UT on 14 September 2001. As in the previous case, all Cluster satellites have observed the TCS (see Figure 2a) and for definiteness' sake we used the data provided by satellite C1 and C2. The current sheet has **LMN** vectors **L** = [−0.9773,0.1062,0.1836], **M** = [0.0429,−0.7488,0.6614], **N** = [0.2077,0.6543,0.7272] for C1 and **L** = [−0.9725,0.1359,0.1892], **M** = [0.0122,−0.7814,0.6239], **N** = [0.2326,0.6090,0.7583] for C2. The behavior of *B*_*L*_ components (for both satellites) is presented in Figure 2b.

The distributions of electric currents, calculated by two methods, are presented in Figure 2f. Similar to case 1, estimation of *ℓ* at the level of 0.5max(*j*_*M*_(*z*)) gives *ℓ*_tim_=1200 km and *ℓ*_rec_=900 km.

For the calculation of the magnetic scaling $\tilde{Z}=\ell /\rho_{T}$, we compute the thermal Larmor radius for the considered case. The magnetic field measured on different sides of the TCS is |*B*|=[|*B*|^before^,|*B*|^after^] = [22,17] nT (see in Figure 2c). However, the behavior of temperature during the TCS crossing is rather complicated (see in Figure 2d). Before entering into the TCS, the temperature has an approximately constant value of 0.8 keV. In the process of the TCS crossing the temperature increases up to 2 keV and keeps its value during 100 s after the crossing. After that, the temperature decreases sharply down to 1.2 keV (after 400 s). Probably, after leaving the TCS the satellites appear in a different plasma, which, as a source of incoming protons, also influences the properties of the TCS. Therefore, we consider in our calculations the time interval from 0 s to 400 s. The corresponding Larmor radii for this TCS are $\rho_{T}=[\rho_{T}^{\text{before}},\rho_{T}^{\text{after}}]=[140,270]$ km. Similar to case 1, we define the average magnetic scaling $<\tilde{Z}>=<\ell >/\rho$ using the corresponding averaged values for the TSC's half thickness and Larmor radius. That gives us $<\tilde{Z}>=5.25$.

The 1‐D distribution functions *f*(*E*) for the considered case are presented in Figure 2e for both sides of the TCS. The corresponding values of averaged energy are $\tilde{E}=[{\tilde{E}}^{\text{before}},{\tilde{E}}^{\text{after}}]=[5.7,\;5.8]$ keV. That finally gives the total averaged *μ* scaling of the TCS <*Z*_*μ*_>=6.2, which is close to the above estimated magnetic scaling $<\tilde{Z}>=5.25$.

### 2.3 Case 3

**Figure 3:**
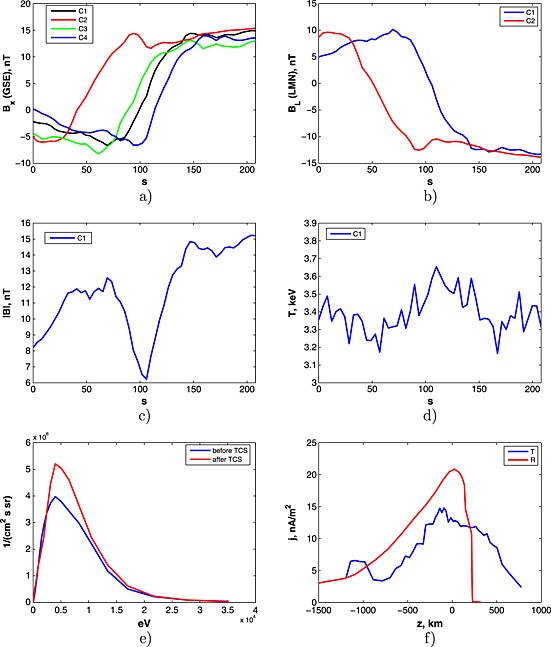
Cluster observations at 15:28:30/15:32:00 UT on 12 August 2001: (a) magnetic field (*x* component) measured by C1, C2, C3, and C4 satellites; (b) *B*_*L*_ ‐ components calculated for C1 and C2; (c) the total magnitude of magnetic field seen by C1; (d) plasma temperature according to C1 measurements; (e) 1‐D energy spectrogram measured by C1 before and after TCS; (f) the recalculated electric current profiles calculated by timing (blue line) and reconstruction (red line) methods.

The event of the TCS crossing in case 3 has start time/end time at 15:28:30/15:32:00 UT on 12 August 2001. All Cluster satellites C1,C2,C3, and C4 observed the TCS (see Figure 3a). For definiteness' sake, we used in the calculations the data provided by satellites C1 and C2. The current sheet has LMN vectors **L** = [−0.9216,−0.2515,0.2955], **M** = [−0.3466,0.1909,−0.9184], **N** = [0.1745,−0.9488,−0.2631] for C1 and **L** = [−0.9115,−0.2537,0.3238], **M** = [−0.4109,0.5986,−0.6876], **N** = [−0.0193,−0.7598,−0.6499] for C2. The behavior of *B*_*L*_ components (for both satellites) is presented on Figure 3b.

The distributions of electric currents along the running coordinate calculated by two methods are presented in Figure 3f. Similar to the previous cases, estimation of *ℓ* at the level of 0.5max(*j*_*M*_(*z*)) gives *ℓ*_tim_=1100 km and *ℓ*_rec_=900 km.

To compute the thermal Larmor radius for the considered case, we take the measure on different sides of the TCS values of the magnetic field |*B*|=[|*B*|^before^,|*B*|^after^] = [12,14.5] nT (see Figure 3c). The temperature behavior during the TCS crossing is not as complicated as in case 2. It has an approximately constant value *T* = 3.3 keV (see Figure 3d). Therefore, the Larmor radii for this TCS are $\rho_{T}=[\rho_{T}^{\text{before}},\rho_{T}^{\text{after}}]=[490,400]$ km with the resulting average value *ρ* = 445 km. That finally gives the average magnetic scaling $<\tilde{Z}>=2.25$.

The 1‐D distribution functions *f*(*E*) for the considered case are presented in Figure 3e for both sides of the TCS. The corresponding values of averaged energy are $\tilde{E}=[{\tilde{E}}^{\text{before}},{\tilde{E}}^{\text{after}}]=[8.0,\;7.97]$ keV. That results in the total averaged *μ* scaling of the TCS <*Z*_*μ*_>=4.3.

This case is particularly interesting because of some disagreement in the estimations of $<\tilde{Z}>=2.25$ and <*Z*_*μ*_>=4.3 (by a factor 2).

One of the possible explanations is that the satellites might oscillate inside the TCS (in frame of LMN system) while gradually leaving it. The specific behavior of magnetic filed magnitude (see Figure 3c) may be considered as a confirmation for such an assumption. In particular, an oscillatory increasing magnetic field is seen by the satellites. In such a situation, the estimated value of *ℓ* may be not sufficiently precise. Another explanation could be that both types of proton populations, i.e., *I*_*z*_=const and *μ* = const protons, equally contribute the main current in the TCS. In this case the scaling of the TCS should be between the corresponding *Z*_*I*_ and *Z*_*μ*_. To check this possibility, we calculate additionally the TCS scaling in case of *I*_*z*_=const protons. We use the following asymptotic expression *Z*_*I*_=(*V*_*T*_/*V*_*D*_)^1/3^. It corresponds the assumption of *V*_*D*_≫*V*_*T*_ [*Sitnov et al.*, 2000]. Following *Sasunov et al.* [2015], we define *V*_*T*_ and *V*_*D*_ in terms of *T* and $\tilde{E}$, respectively, and finally obtain $Z_{I}={\left(T/2\tilde{E}\right)}^{1/6}$. For the particular parameters of the considered case, i.e., $\tilde{E}=8$ keV, *T* = 3.4 keV, this scaling is *Z*_*I*_=0.77.

Note that both *Z*_*I*_ and *Z*_*μ*_ scalings do not give values close to observed one: $\tilde{Z}$. However, in view of the proposed above assumption on equal contribution of both types of protons to the main current in the TCS, its scaling may be defined as an average of the corresponding scalings: <*Z* >= (*Z*_*μ*_+*Z*_*I*_)/2 = (4.3 + 0.77)/2 = 2.53. The obtained value is close to the observed scaling $<\tilde{Z}>=2.25$, which might confirm the validity of the made assumption.

## 3 Conclusion

In this paper we present the study of three TCS crossings observed by Cluster in 2001. It turns that the different methods for determination of the running coordinate −*z* give sometimes different internal structure of the TCS, such as bifurcation (see Figures 1f and 2f). Therefore, it is necessary to find additional criteria to identify the internal structure of TCS.

We note that different dynamic regimes of the TCS‐forming protons, which correspond to *I*_*z*_=const and *μ* = const conditions, respectively, result in different scaling. That enables to interpret the measured geometry features of the real TCSs without making additional assumptions regarding magnetic field profile and the corresponding renormalization. In particular, regarding the considered cases, the proposed *Z*_*μ*_ may be used to predict the thickness of the TCS and, by comparing it with the thickness obtained from the spacecraft measurements, to judge on the type of dynamics of the current carriers which create the TCS. In that respect the results of *Runov et al.* [2005] and *Runov et al.* [2006] may indicate that in the most observed cases the TCSs are created by *μ* = const protons. However, to confirm this preliminary conclusion, it will be necessary to make an additional statistical comparative study of the $<\tilde{Z}>$ and *Z*_*μ*_ scalings.

The proposed concept of two types of the TCS‐forming proton populations may be also used to construct a scenario for the adiabatic compression of a TCS without changing of magnetic field value. For instance, if the initial TCS is created by protons with *μ* = const, then a graduate change of the protons' dynamic regime toward the *I*_*z*_=const type will result in the decreasing *ℓ* due to the change of the scaling from *Z*_*μ*_>1 to *Z*_*I*_<1. The experimental check of this idea is a subject for further study. It concerns also the issue of the stability of such TCSs and their related transformations.
